# Effect of KOROPASS, an extruded jack bean (*Canavalia ensiformis*)-derived supplement, on productivity and economic performance of beef cattle

**DOI:** 10.14202/vetworld.2020.593-596

**Published:** 2020-03-29

**Authors:** Bambang Waluyo Hadi Eko Prasetiyono, Agung Subrata, Widiyanto Widiyanto

**Affiliations:** Department of Animal Science, Faculty of Animal and Agricultural Sciences, Diponegoro University, Semarang, Central Java, Indonesia

**Keywords:** beef cattle, extruded jack bean, feed utilization, growth

## Abstract

**Aim:**

This study evaluated the effect of feeding a graded amount of extruded jack bean (*Canavalia ensiformis*) on nutritional status, production performances, and economic performance of beef cattle.

**Materials and Methods:**

The supplement called “KOROPASS” was prepared from the extruded jack bean (according to the extrusion heating process). Sixteen male Friesian-Holstein crossbred cattle were divided into four groups and fed on KOROPASS as per the regimen: R_0_ (total mixed ration [TMR] without KOROPASS), R_1_ (TMR supplemented with 3% KOROPASS), R_2_ (TMR supplemented with 6% KOROPASS), and R_3_ (TMR supplemented with 9% KOROPASS). The *in vivo* experiment lasted 44 days. TMR contained 12% crude protein and 60% total digestible nutrient. The consumption and digestibility of dry matter (DM), organic matter (OM), and total protein (TP), feed efficiency, average daily gain, and income over feed cost (IOFC) were evaluated.

**Results:**

KOROPASS supplementation significantly increased (p<0.05) beef cattle consumption of DM (from 7.83 [R_0_] to 8.33 [R_1_], 8.91 [R_2_], and 9.69 kg/day [R_3_]), OM (from 6.72 to 7.17, 7.69, and 8.38 kg/day, respectively), and TP (from 892 to 1020, 1182, and 1406 g/day, respectively). The elevated levels of KOROPASS significantly increased (p<0.05) digestibility in terms of the levels of DM (from 42.9 [R_0_] to 50.6 [R_1_], 58.0 [R_2_], and 63.6% [R_3_]), OM (from 54.3 to 59.6, 66.3, and 70.6%, respectively), and TP (from 65.0 to 67.1, 75.0, and 80.7%, respectively). Dietary supplementation of KOROPASS significantly increased (p<0.05) metabolizable protein, average daily weight gain, and feed efficiency of beef cattle. Finally, dietary KOROPASS supplementation, especially at 9%, resulted in the highest (p<0.05) IOFC value of beef cattle.

**Conclusion:**

Dietary supplementation of KOROPASS improved feed utility, as reflected by the increase in consumption and digestibility of DM, OM, and TP. Further, KOROPASS supplementation improved feed efficiency, growth, and economic performance of beef cattle. The findings indicate the potential value of KOROPASS as a feed supplement for beef cattle.

## Introduction

The increasing demand for beef in Indonesia has outpaced local beef production. In 2018, Indonesia had to import 400,000 heads of beef cattle and 93,000 tons of beef [1]. Low livestock productivity, which leads to low economic performance, is one of the main factors inhibiting the expansion of cattle farming in Indonesia. The low quality and quantity of the feed consumed by beef cattle are linked to their low growth features. In general, the inability of farmers to provide standard feed for beef cattle is mainly caused by the high prices of quality feed, especially feed ingredients that contain high levels of protein, such as soybeans, which are still imported and are not affordable for farmers.

Indonesia has diverse and readily available vegetation, such as jack bean (*Canavalia ensiformis*), that can be a source of the protein needed for feed supplementation [2]. However, the dietary incorporation of jack bean in beef cattle feed has not been explored.

Jack bean contains relatively high levels of protein (34.6%) [3]. However, the rate of protein degradation in the rumen of beef cattle is also high (approximately 56.7%) [2]. In addition, the hydrogen cyanide content of jack beans is approximately 11.05 mg/100 g, which may harm the rumen ecosystem of cattle [4]. An *in vitro* study reported that the extrusion heating process could improve the rumen-protected protein (RPP) of jack bean [2]. The authors described that extrusion heating increased the RPP level from 43.35% to 59.16% and decreased the rumen level of NH_3_ from 5.28 mM to 2.71 mM. In general, heating of protein-rich feed ingredients using extrusion heating techniques results in the Maillard reaction (browning reaction) between the reducing sugars and protein [5]. The reaction protects the extruded feedstuffs from degradation in the rumen and, therefore, increases the availability of nutrients for absorption in the small intestine [6,7]. This would facilitate the efficiency of protein biosynthesis, which is reflected in the improved growth of beef cattle. To the best of our knowledge, the use of extruded jack bean to improve the growth, productivity, and economic performance of beef cattle has never been reported.

In the present study, jack bean was used as the source of RPP and was extruded before incorporation into a corncob-based total mixed ration (TMR). The effects of feeding a graded level of the extruded jack bean on nutritional status, growth, feed cost and income over feed cost of beef cattle were investigated.

## Materials and Methods

### Ethical approval

The *in vivo* experiment was approved by the animal ethics committee of the Faculty of Animal and Agricultural Sciences, Diponegoro University (No. 3084/UN7.5.5/KP/2017, 22 May 2017).

### Materials

Jack bean was purchased from Temanggung Regency, Central Java Province, Indonesia. The jack bean-based preparation designated KOROPASS was obtained following a previously described extrusion heating process using jack bean [2].

### Experimental design

Sixteen male Friesian-Holstein crossbred cattle (approximately 1.5 years old) were divided according to body weight into four treatment groups (n=4 per group). The cattle were placed in individual pens disinfected and treated with albendazole. The treatment groups included TMR without KOROPASS as control (R_0_), and TMR supplemented with 3% KOROPASS (R_1_), 6% KOROPASS (R_2_), and 9% KOROPASS (R_3_). The quantity of TMR was 9.11, 9.41, 9.78, and 10.3 kg/day (as-fed basis) for R_0_, R_1_, R_2_, and R_3_, respectively. The quality of KOROPASS used to supplement TMR was 0, 0.27, 0.56, and 0.89 kg/day (as-fed basis) for R_0_, R_1_, R_2_, and R_3_, respectively. The *in vivo* experiment lasted for 44 days. The cattle were in the growth phase and were very responsive to protein supplementation. The 44-day duration of the experiment was considered sufficient to study the effect of KOROPASS on the performance parameters, as previously conducted by Prasetiyono *et al*. [8]. All the beef cattle were adapted to TMR for 2 weeks before the *in vivo* experiment. The ingredients and chemical composition of TMR are listed in Table-1. The ration contained 12% crude protein and 60% total digestible nutrient (TDN). The consumption and digestibility of dry matter (DM), organic matter (OM), and total protein (TP); feed efficiency; and average daily gain were determined as previously described [9]. In addition, income over feed cost (IOFC) was also measured based on Prasetiyono *et al*. [8].

**Table-1:** Ingredients and nutrient composition of TMR.

Ingredients	Proportion (%)
Corncob	20.0
Mineral mix “StV”	1.00
Salt	1.00
Cassava waste	10.0
Pollard	21.0
Molasses	7.00
Calcium carbonate	1.00
Corn straw	5.00
Degraded protein supplement (Go Pro)	2.00
Nutshell	6.00
Corn gluten feed	26.0
Nutrient composition	
Dry matter	86.0
Ash	7.18
Crude protein	12.2
Ether extract	1.92
Crude fiber	18.0
Total digestible nutrient	60.0
Ca	0.90
P	0.60

TMR=Total mixed ration

### Statistical analysis

The data collected were analyzed using analysis of variance on the basis of a randomized complete block design [10].

## Results and Discussion

In this study, the effect of block was not significant, and therefore the block effect was not considered. KOROPASS supplementation as the source of RPP significantly increased (p<0.05) the consumption of DM, OM, and TP in the beef cattle (Table-2). The findings suggest that dietary supplementation by KOROPASS improved the palatability of TMR derived from corncobs, an agricultural by-product. The increased protein content of the KOROPASS supplemented TMR seemed to be responsible for the increased palatability and better feed consumption by the beef cattle. The findings support earlier study which reported that feed consumption can be affected by dietary supplementation, feed quality, and the availability of particular food components, such as protein [11]. Consistent with this, dietary supplementation with urea (non-protein nitrogen) increased feed consumption in beef steers [12]. The increased levels of the KOROPASS supplementation attributed to the increased contents of protein in the rations and thus the improved intake of DM, OM, and TP of beef cattle.

**Table-2:** Effect of KOROPASS supplementation in the TMR on variables measured.

Variables	Treatments				SEM	p-value

	R_0_	R_1_	R_2_	R_3_		
DM consumption (kg/day)	7.83^d^	8.33^c^	8.91^b^	9.69^a^	0.07	<0.05
OM consumption (kg/day)	6.72^d^	7.17^c^	7.69^b^	8.38^a^	0.07	<0.05
TP consumption (g/day)	892^d^	1,020^c^	1,182^b^	1,406^a^	0.04	<0.05
DM digestibility (%)	42.9^d^	50.6^c^	58.0^b^	63.6^a^	1.16	<0.05
OM digestibility (%)	54.3^d^	59.6^c^	66.3^b^	70.6^a^	0.94	<0.05
Crude protein digestibility (%)	65.0^b^	67.1^b^	75.0^a^	80.7^a^	1.86	<0.05
Metabolizable protein (%)	49.0^b^	52.2^b^	55.0^b^	65.2^a^	3.10	<0.05
Average daily gain (kg/day)	0.72^c^	0.83^c^	0.99^b^	1.24^a^	0.05	<0.05
Feed efficiency (%)	9.50^c^	10.24^bc^	11.53^ab^	13.14^a^	0.51	<0.05
Feed cost (IDR/head/day)	26,403^d^	29,177^c^	32,274^b^	36,222^a^	265	<0.05
IOFC (IDR/head/day)	6832^b^	8888^b^	13,151^b^	20,933^a^	1996	<0.05

Numbers with different letters on the same row show difference at p<0.05. “a” represents the highest value, and “d” represents the lowest values. Price (at the time of study) per kg of TMR=IDR 2900, KOROPASS=IDR 7000, Beef cattle=IDR 46,000 (price per kg live weight). DM=Dry matter, OM=Organic matter, TP=Total protein, IOFC=Income over feed cost, TMR=Total mixed ration, IDR=Indonesian rupiah (Indonesian currency), SEM=Standard error of the mean

The degree of DM and OM digestibility increased significantly (p<0.05) in relation to the increased KOROPASS content in the TMR (Table-2). It is likely that dietary supplementation with the protein-rich KOROPASS increased rumen microbial proliferation and activity, leading to the increased fermentation rate in the rumen [13], which, in turn, may contribute to improving the digestibility of DM and OM in cattle [13,14]. In addition, increased KOROPASS supplementation significantly improved the digestibility of crude protein (p<0.05). Moreover, KOROPASS supplementation increased the availability and utilization of protein in the intestine, as most of the jack bean protein could escape ruminal fermentation. These findings indicate that the KOROPASS could increase the supply of nitrogen to rumen microbes and support the findings of an earlier [15].

Dietary supplementation of KOROPASS significantly increased (p<0.05) the metabolizable protein of cattle (Table-2). Theoretically, the metabolizable protein is the total amount of protein available for digestion in the post-rumen digestive tract, which includes feed protein that escaped rumen degradation as well as microbial protein (bacterial biomass) [16]. Therefore, the increased metabolizable protein in the cattle fed on KOROPASS supplemented feed might be contributed by the increased microbial protein (bacterial biomass) as well as protein from the KOROPASS escaping from rumen fermentation.

KOROPASS supplemented TMR significantly increased (p<0.05) the average daily weight gain of beef cattle (Table-2). The results imply that KOROPASS supplementation increased tissue biosynthesis in beef cattle. Several factors may contribute to the improved daily gain, such as the increased consumption and digestibility of DM, OM, and protein. Furthermore, the increased metabolizable protein is likely to increase the growth performance of cattle. Protein is the most important nutrient for tissue biosynthesis. Thus, the increased intake and digestibility of protein is expected to positively affect the daily gain of cattle [13,17]. Energy is another factor that may determine the rate of growth of cattle [18]. The increases in DM and OM consumption and digestibility in the KOROPASS treated cattle could be attributed to the increased energy supply for growth.

Dietary supplementation of KOROPASS was associated with significantly improved (p<0.05) feed efficiency of the cattle. In accordance with our findings, Uddin *et al*. [13] documented that protein supplementation may have been associated with increased nutrient utilization and growth and thus improved feed efficiency of cattle.

IOFC is used to evaluate the profitability and sustainability of cattle farms. In the present study, dietary supplementation with KOROPASS, especially at 9%, resulted in a significantly higher (p<0.05) IOFC value of the cattle. The measured parameters convincingly demonstrated that RPP derived from KOROPASS increased feed utilization and efficiency, as well as growth performance of cattle. Jack bean is abundantly available in Indonesia. However, it remains underutilized and unexplored as an affordable feed component for cattle. Given its’ relatively low price and high nutritional value, the use of extruded jack bean as an RPP source is an attractive option to improve the IOFC of cattle farms.

## Conclusion

Dietary supplementation of KOROPASS jack bean-based RPP improved feed utility, as reflected by the increased consumption and digestibility of DM, OM, and TP, and improved feed efficiency, growth, and economic performance of beef cattle.

## Authors’ Contributions

BWHEP designed, carried out the experiment, and drafted the manuscript; AS and WW carried out the *in vivo* experiment, conducted data analysis, and revised the manuscript. All authors read and approved the final manuscript.
